# Functional Characterization of Enone Oxidoreductase Orthologs Highlights Hidden Furaneol Biosynthetic Capacity in Apples

**DOI:** 10.1021/acs.jafc.6c02103

**Published:** 2026-04-22

**Authors:** Chan Li, Katharina Jahr, Yujun Sun, Benedikt Johannes Thalhammer, Julia Schweppe, Xiran Wang, Timothy D. Hoffmann, Thomas Hoffmann, Wilfried Schwab

**Affiliations:** Biotechnology of Natural Products, School of Life Sciences, 9184 Technical University of Munich, Freising 85354, Germany

**Keywords:** enone oxidoreductase, quinone oxidoreductase, strawberry, apple, pineapple, Furaneol, 4-hydroxy-5-methyl-2-methylene-3(2*H*)-furanone

## Abstract

4-Hydroxy-2,5-dimethyl-3­(2*H*)-furanone (HDMF, Furaneol) is an important flavor compound in many fruits. It is formed from the precursor 4-hydroxy-5-methyl-2-methylene-3­(2*H*)-furanone (HMMF) by an enone oxidoreductase (EO), which has previously been characterized only in strawberry, tomato, and mango. Here, we heterologously produced EO orthologs from pineapple, kiwi, and grape, three species known to accumulate HDMF, as well as from apple and crab apple, which do not produce this compound. All candidates were expressed in fruit tissue and exhibited *o*-quinone oxidoreductase activity, reducing 9,10-phenanthrenequinone and various *o*-quinones. Importantly, all enzymes catalyzed HDMF formation, including those from apple species. The absence of HDMF accumulation in apples was attributed to the lack of the natural precursor HMMF and low levels of active EO, while HDMF was rapidly converted to its glucoside. The conserved *o*-quinone oxidoreductase activity across all EO orthologs suggest that fruit EOs involved in HDMF biosynthesis evolved from a broader quinone oxidoreductase family.

## Graphical abstract

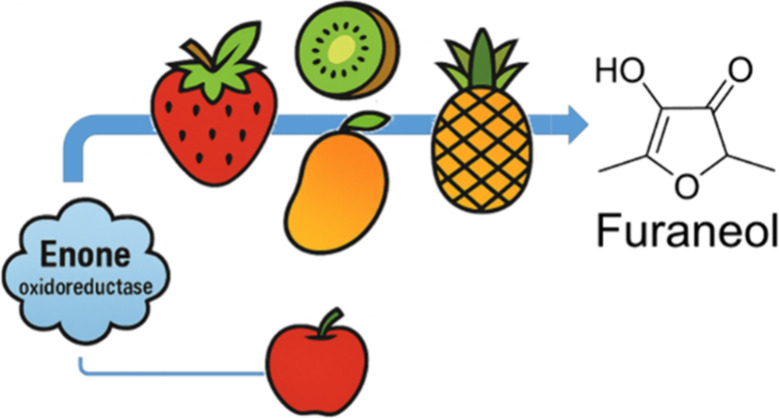

## Introduction

Fruits, as essential components of the human diet, are appreciated for their distinctive textures and diverse aromatic profiles. Aroma is one of the most important indicators of the quality and sensory characteristics of fresh fruit.^1^ The unique flavor of any given fruit species results from the combined diversity and concentration of numerous volatile compounds. Among these, 4-hydroxy-2,5-dimethyl-3­(2*H*)-furanone (HDMF, Furaneol), originally identified as a product of the Maillard reaction,^2^ is well-known for its extremely low odor threshold and its high abundance in strawberries.^3^ HDMF was first isolated from pineapples^4^ and subsequently detected in strawberries, mangoes, tomatoes, kiwis, wine grapes, and many other fruits.^5,6^

At low concentrations, HDMF exhibits a strawberry-like aroma, whereas at higher concentrations it imparts a sweet, caramel-like flavor. Its caramel-like flavor is associated with a planar enol-oxo group in a cyclic dicarbonyl derivative, which is capable of forming strong hydrogen bonds.^7^ To date, the biosynthesis and metabolism of HDMF in strawberry have been extensively investigated, but remain incompletely understood. Radiotracer studies and substances labeled with stable isotopes demonstrated that d-fructose-1,6-diphosphate (FDP) is converted by an as-yet-unknown enzyme to 4-hydroxy-5-methyl-2-methylene-3­(2*H*)-furanone (HMMF), which serves as a natural precursor for *Fragaria × ananassa* quinone oxidoreductase (FaQR) that catalyzes the final biosynthetic step to HDMF.^8–12^

HDMF can undergo several downstream metabolic transformations in strawberry. It can be converted into its methyl ether 2,5-dimethyl-4-methoxy-3­(2*H*)-furanone (DMMF), by *Fragaria ananassa* O-methyltransferase (FaOMT).^1,12–14^ Alternatively, it can be stabilized through glycosylation to form HDMF β-d-glucoside by UDP-dependent glycosyltransferases.^15^ This glucoside is then further modified into its malonylated derivative by malonyltransferase (FaMAT).^16–18^ The enzyme capable of reducing HMMF to HDMF was initially named FaQR, based on its sequence similarity to known quinone oxidoreductases and its catalytic activity toward the surrogate substrate 9,10-phenanthrenequinone (PQ).^10^ It was later renamed *Fragaria × ananassa* enone oxidoreductase (FaEO) because it efficiently catalyzes the reduction of the exocyclic unsaturated bond of HMMF and its derivatives.^7^

Structural analysis of FaEO revealed that the enzyme exhibits a narrow substrate spectrum, as only planar enones can enter its active site.^11^ The transfer of the *4R*-hydride of NAD­(P)H to the exocyclic carbon double bond of the substrate results in reduction through a formal 1,4-hydrogen addition.^11^ In parallel, transcriptional regulation studies showed that an ERF–MYB complex, consisting of FaERF#9 and FaMYB98, activates the *FaEO* promoter and thereby up-regulates HDMF biosynthesis in strawberry.^19^

Although HDMF and its derivatives have been detected in a wide range of fruits, research on their biosynthesis is still limited. Apart from the partial functional characterization of enone oxidoreductases (EOs) in strawberry, tomato, and mango, the pathways responsible for HDMF formation and the roles of related EOs in other fruits remain largely unexplored. Although HDMF is an established contributor to the aroma of numerous fruits, there have been no reports to date describing the presence of HDMF or its derivatives in apple, despite its taxonomic proximity to strawberry within the Rosaceae family.^20^

Substrate inhibition (SI) describes an enzymological phenomenon in which an enzyme’s catalytic activity is reduced or fully inhibited once the substrate concentration exceeds a certain threshold.^21^ Recently, a new substrate inhibition model (asymmetric cooperativity) has been proposed, which perfectly explains the substrate inhibition of a monomeric bisubstrate enzyme (*Nb*UGT72AY1) with only one binding site for each substrate.^22^ They found that the binding order of the acceptor substrate (scopoletin, S) and the donor substrate (UDP-glucose, U) to the enzyme (E) affects the enzymatic activity. In short, although the binding is random (E–S-U or E–U-S), the reaction occurs only if a specific order is followed (E–U-S). This model consolidates two classical models (random and sequential ordered) for substrate binding in a bisubstrate enzyme, which provides more possibilities for further research on substrate inhibition.^22^

To enable comparison, we cloned and expressed eight *EO*s from six fruit species known to accumulate HDMF and from two apple species that do not produce this compound and its derivatives. Heterologous expression and functional characterization revealed that all candidates exhibited quinone oxidoreductase (QR) activity and were able to reduce various *o*-quinones. Moreover, all were capable of synthesizing HDMF. Further experiments indicated that the lack of HMMF as a natural precursor, together with the low abundance of active EO in apple fruit, underlies the absence of HDMF accumulation.

## Materials and Methods

### Chemical

*O*-Chloranil, 9,10-phenanthrenequinone (PQ), 3-mercaptobenzoic acid, HDMF, and homofuraneol were obtained from Sigma-Aldrich (Taufkirchen, Germany). 1,2-Naphthoquinone and 3,5-di*tert*-butyl-*o*-benzoquinone were purchased from Thermo Fisher Scientific (Dreieich, Germany). 4,5-Dimethoxy-*o*-benzoquinone was sourced from Biosynth (Staad, St. Gallen, Switzerland). Pectinase was obtained from AB Enzymes (Feldberg, Germany), and D-fructose-1,6-diphosphate, NADH, and NADPH from Carl Roth (Karlsruhe, Germany). PVPP (polyvinylpolypyrrolidone) was purchased from Erbslöh Geisenheim (Geisenheim, Germany).

### Search for Enone Oxidoreductases in Candidate Fruits

BLAST searches (https://blast.ncbi.nlm.nih.gov/Blast.cgi) were performed against the genomes of several fruit species using the full-length amino acid sequence of FaEO (AAO22131.1), SlEO (NP_001296292.1), and MiEO (AFJ53076.1) as the query sequences. Due to their high sequence similarity to the queried EOs, the proteins MdEO (XP_008389642.1) from *Malus domestica*, MsEO (XP_050103939.1) from *Malus sylvestris*, AccEO (PSR84843.1) from *Actinidia chinensis*, AncEO (XP_020106159.1) from *Ananas comosus*, and VvEO (XP_003631255.1) from *Vitis vinifera* were selected for subsequent analysis (Figure 1).

**Figure 1:**
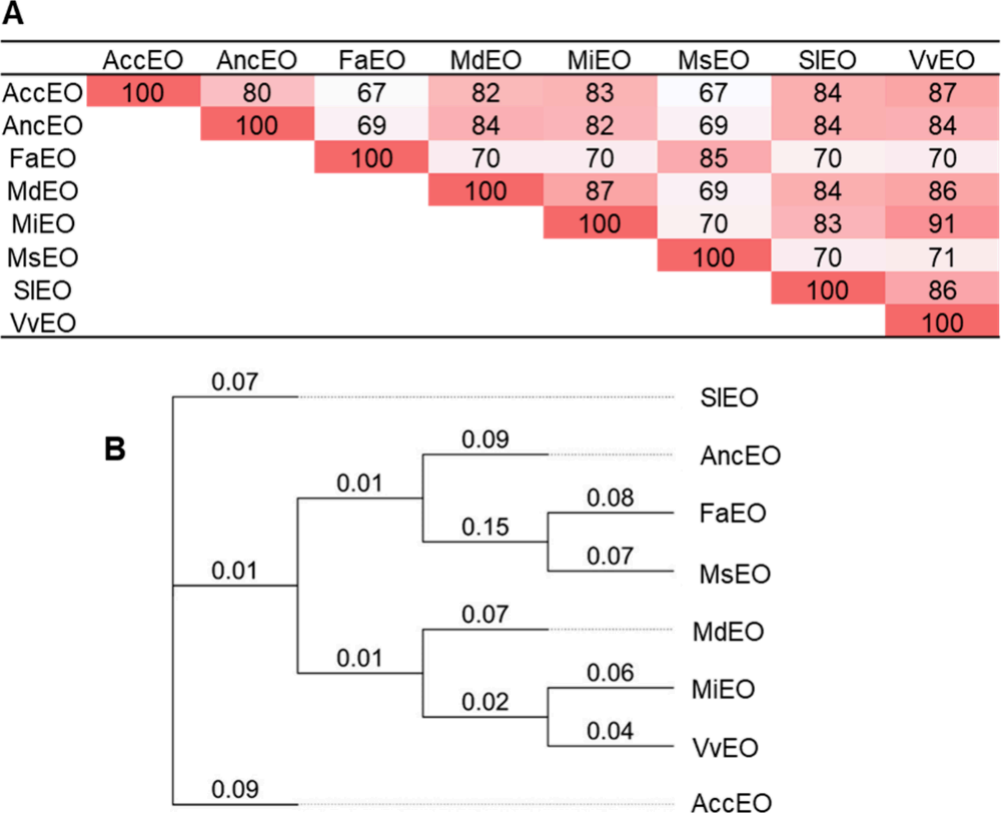
Protein sequence analysis of EO enzyme orthologs from different fruit. (A) Protein sequence identity of EO candidates: FaEO from *Fragaria x ananassa* (Q84 V25), SlEO from *Solanum lycopersicon* (NP_001296292), MiEO from *Mangifera indica* (AFJ53076), VvEO from *Vitis vinifera* (RVX10457 or XP_003631255), AncEO from *Ananas comosus* (XP_020106159), AccEO from *Actinidia chinensis* (PSR84843), MdEO from *Malus domestica* (XP_008389642), and MsEO from *Malus sylvestris* (XP_050103939). Sequences lacking the predicted signal peptides were used for analysis (Supplementary Table S3). (B) Phylogenetic tree constructed with the eight sequences. Sequences were aligned with Geneious 5.6.7 using the Geneious alignment tool with default values. The tree was built by the Neighbor-Joining method with no outgroup and 1000 replicates. The genetic distance model was Jukes-Cantor. The numbers indicate substitutions per site at amino acid level.

### Cloning of the Candidate EOs

Genewiz, Leipzig, Germany (www.genewiz.com) after codon optimization for *Escherichia coli*, synthesized the genes *MiEO*, *MdEO*, *MsEO*, *AccEO*, *AncEO*, and *VvEO* (Supplementary Table S1). The genes were ligated via *NcoI* at the 5′ end and the *XhoI* site (*NotI* for *AncEO*) at the 3′ end into the pET-29a (+) vector, according to the manufacturer’s instruction (Merck, Darmstadt, Germany). The resulting recombinant plasmids were amplified in *E. coli* NEB 10β cells (New England Biolabs, Frankfurt, Germany) for selecting positive clones. Following sequencing, the genes were transformed into *E. coli* BL21­(DE3) plysS host strain for expression, according to the manufacturer’s instruction (Thermo Fisher Scientific, Waltham, MA, USA).

### RNA Extraction and Quantitative Real-Time PCR (qPCR) Analysis

Total RNA was extracted from all fruit samples using the RNeasy Plant Mini Kit (Qiagen, Venlo, The Netherlands). The cDNA synthesis for qPCR was performed using the iScript cDNA Synthesis Kit (Bio-Rad, Hercules, CA, USA). The qPCR primers were designed by NCBI tools (Supplementary Table S2). For qPCR analysis, cDNA was diluted to 50 ng/μL in sterile RNase-free water. Each reaction (20 μL total volume), contained 2 μL of diluted cDNA and 400 nmol/L of each primer, using the SensiFAST SYBR Hi-ROX Mix (Meridian Bioscience, Cincinnati, OH, USA). Cycling conditions followed the manufacturer’s instruction. For each fruit candidate, a reference gene previously validated for stable expression was used for normalization (Supplementary Table S2).

### Protein Expression and Purification

Heterologous expression of the candidate proteins was carried out in *E. coli* BL21­(DE3) plysS in LB Medium with 25 μg/mL kanamycin and 34 μg/mL chloramphenicol. After overnight preculturing at 37 °C with shaking at 150 rpm, 10 mL of the preculture was inoculated into 1 L main culture, which was grown to an OD_600_ = 0.8–1.0. The culture was supplemented with 1 mM isopropyl-β-d-thiogalactopyranoside (IPTG) and incubated overnight (48 h for MiEO) at 18 °C and 150 rpm. Cells were harvested by centrifugation and stored at −80 °C. Recombinant proteins with an *C*-terminal His-tag were purified with ProBond Resin (NOVEX) following the manufacturer’s instructions with slight optimization (Thermo Fisher Scientific, Waltham, MA, USA). Briefly, cell pellets were resuspended and disrupted by sonication in 1x His-wash/bind buffer containing 30 mM imidazole and 200 μL of 100 mM phenylmethylsulfonyl fluoride (PMSF). Following centrifugation, the clarified supernatant was incubated overnight at 4 °C with the affinity resin. The resin was washed with increasing concentrations of imidazole (30 mM, 40 mM, and 50 mM) in His wash/bind buffer, and the bound proteins were eluted using His elution buffer containing 500 mM imidazole. Protein purity was assessed by sodium dodecyl sulfate-polyacrylamide gel electrophoresis (SDS-PAGE) (Supplementary Figure S1), and protein concentration was determined by comparison to a series of bovine serum albumin (BSA) standards run on the same gel. The target protein was exchanged from the imidazole-containing elution buffer into 20 mM Bis-Tris buffer (pH 7.5) using a PD-10 desalting column and stored at −20 °C (Merck, Darmstadt, Germany).

### Substrate Screening and Kinetics Analysis

The *o*-quinones used for substrate screening included *o*-chloranil, 1,2-naphthochinon, 3,5-di*tert*-butyl-*o*-benzoquinone, 4,5-dimethoxy-*o*-benzoquinone, and 9,10-phenanthrenequinone (PQ). For substrates converted by the enzymes, the kinetic data were measured. Substrate concentrations from 0.5 to 200 μM were added to assay mixtures containing 1–3 μg of purified enzyme and 360 μM NADH in 200 μL of Na_2_HPO_4_/NaH_2_PO_4_ buffer (0.1 M, pH 7). In determining the NADH kinetics, we used PQ at a saturating concentration of 120 μM. Enzyme activity was monitored by measuring the decrease in absorbance at 340 nm corresponding to NADH consumption with ClarioStar microplate reader (BMG Labtech, Ortenberg, Germany). Michaelis–Menten parameters, *K*_*m*_ and *v*_*max*_ were determined by fitting the data to the classical Michaelis–Menten equation using OriginPro software (www.originlab.com). All measurements represent the mean of three independent replicates.

### In Vitro Enzymatic Assay and HDMF Extraction

Fifteen micrograms of candidate EO purified from *E. coli* were incubated with 40 mg D-fructose-1,6-diphosphate and 2 mg NADH in 20 mM Bis-Tris buffer (pH 7.5) for 15 h at 30 °C and 150 rpm. After incubation, 100 μL of 0.05 mg/mL homofuraneol standard was added to the reaction solution, which was frozen at −80 °C to terminate the reaction. HDMF was extracted from assay samples using Chromabond C18ec solid phase extraction columns (Macherey-Nagel, Düren, Germany) according to the manufacturer’s instructions. Briefly, the columns were conditioned with six column volumes of methanol followed by six column volumes of water. Subsequently, 1.1 mL of the reaction mixture was loaded onto the column. After washing with 1 mL water, the column was dried under vacuum and eluted using six column volumes of diethyl ether. The eluate was collected, residual water was removed with anhydrous Na_2_SO_4_, 50 μL water was added, and the organic solvent was removed under a gentle stream of nitrogen. HDMF was quantified by HPLC-UV-MS analysis.

### Furanone Quantification by HPLC-UV-MS Analysis

HDMF, HDMF β-d-glucoside, and HDMF β-d-glucoside-malonate were quantified with an HPLC system equipped with a Phenomenex Luna C18(2) column (150 mm long × 2.0 mm inner diameter, particle size 5 μm, 100 A; Phenomenex, Aschaffenburg, Germany). An Agilent 6340 Ion Trap mass spectrometer (Agilent Technologies, Santa Clara, CA, USA) connected to an Agilent 1200 HPLC system equipped with a capillary pump and a diode array detector was utilized. The LC was performed with the following binary gradient system: solvent A, water with 0.1% formic acid, and solvent B, 100% methanol with 0.1% formic acid. The gradient program was as follows: 0–50% B in 3 min; 50–100% B in 3 min; 100% B for 8 min; 100–0% B in 10 s; 0% B for 10 min. The injection volume was 5 μL, and the flow rate was 0.2 mL/min. Furanones were identified by their retention time and quantified using Openlab (Agilent Technologies, Santa Clara, CA, USA) using their UV absorbance at 280 nm.

### HDMF Extraction from Fruit Flesh

Two hundred g of fruit were homogenized with 200 mL of water using a blender, and the homogenate was centrifuged for 30 min at 14,069×*g*. The supernatant was collected after filtration through glass wool. HDMF and its derivatives were isolated by adsorption onto an Amberlite XAD-2 column according to,^12^ with minor modifications. Briefly, the column was conditioned with three column volumes of methanol followed by three column volumes of water, after which the extract was loaded. The column was eluted sequentially with 100 mL each of water, diethyl ether, and methanol. The diethyl ether fraction, containing HDMF, was collected and evaporated, and the residue was adjusted to a final volume of 2 mL with water for HPLC analysis. The methanol fraction, containing HDMF β-d-glucoside and HDMF β-d-glucoside-malonate, was evaporated to dryness and redissolved in 2 mL of water prior to HPLC analysis.

### HDMF Injection into Apples

An aqueous HDMF solution (400 μL; 10 mg/mL) was divided into four equal aliquots and injected into different positions of intact apples. The apples were then incubated for 0, 15, or 48 h. Following incubation, apple tissues were extracted using the same procedure described above for HDMF extraction from fruit flesh.

### Protein Extraction from Fruit Flesh

Five hundred g of ripe fruit were immersed in 200 mM CaCl_2_ for 1 h to stabilize pectins and subsequently washed thoroughly with distilled water. The fruits were juiced using a household press fitted with a nylon net (150 μm). Ten g polyvinylpolypyrrolidone (PVPP) and 5 mL of 100 mM ethylenediaminetetraacetic acid (EDTA) were added to 100 mL of collected juice. The mixture was adjusted to pH 5.0 and centrifuged at 21,191×*g* and 4 °C for 30 min. Pectinase solution (ROHAPECT PTE 100, 7–10 PTF/μL) was added to the resulting supernatant at a concentration of 10 μL per 100 mL. The enzyme preparation had a declared pectin lyase activity of 100 PTF/mg, measured with pectin as substrate at pH 5.8 and 30 °C. One unit of PTF is defined as the amount of enzyme which produces 1 μmol of unsaturated uronide (ε = 5,500 L/(mol cm) at 235 nm) per min under the reaction conditions. The supernatant was then transferred into dialysis tubing with a 6 kDa molecular-weight cutoff and dialyzed overnight at 4 °C against 20 mM Bis-Tris buffer. The supernatant was collected and centrifuged again to remove any residual PVPP. Crude protein was concentrated from the supernatant by Vivaspin spin columns (molecular weight cutoff 30 kDa; Merck, Darmstadt, Germany).

### HMMF Extraction and Detection

Eight hundred microliters of a 6 mM 3-mercaptobenzoic acid solution (70% v/v, ethanol) were divided into four aliquots and injected at different positions into whole apple fruits and strawberries. The treated fruits were stored at room temperature for 3 days. Subsequently, the fruits were subjected to XAD-2 solid-phase extraction, and the diethyl ether and methanol extracts were analyzed for the presence of trapped HMMF using LC-MS. The LC-MS detection method published by^7,10^ was applied. Trapped HMMF was identified as MBS-HMMF isomers based on their retention times, mass spectra, and product ion spectra using DATA_ANALYSIS v.4.0 software (Bruker Daltonik GmbH, Bremen, Germany).

### Kinetic Evaluation of Substrate-Enzyme Complexes Exhibiting Substrate-Inhibition Behavior

The conventional random sequential model for bisubstrate enzymes proposed by^23^ typically assumes functional equivalence between the two branches leading to the ternary complex. However, emerging structural and biochemical evidence indicate that substrate binding triggers ligand-dependent conformational rearrangements within the enzyme, consequently, distinct enzyme–substrate complexes formed via alternative binding branches may exhibit markedly different catalytic properties.^22,24^ Based on the sequential randomness of the two branches, two Hill coefficients (*n* and *x*) were introduced (eq 1),^25^ which describe the unequal homotropic influence on ligand binding in the two branches of a sequential random binding system (*n* ≠ *x*). The associated steady-state kinetic model accounts for cooperative substrate binding, characterized by the Hill coefficients. One consequence of the inequality of the Hill coefficients is substrate inhibition (SI).^22^

$$v=\frac{v_{max}\times \frac{[S{]}^{n}}{K_{D}^{n}}}{1+\frac{[S{]}^{n}}{K_{D}^{n}}+\frac{[S{]}^{x}}{K_{D}^{x}}}$$ 1

If the ternary complexes formed by different binding sequences of two substrates all exhibit different catalytic activities, the equation can be further improved to (eq 2).^22^

$$v=\frac{v_{max}\times \frac{[S{]}^{n}}{K_{D}^{n}}+v_{i}\times \frac{[S{]}^{x}}{K_{D}^{x}}}{1+\frac{[S{]}^{n}}{K_{D}^{n}}+\frac{[S{]}^{x}}{K_{D}^{x}}}$$ 2

*v*_*max*_ represents the maximum velocity when the ternary complex with high catalytic activity dominates. *v*_*i*_ represents the maximum velocity when the ternary complex with low catalytic activity dominates. *K*_*D*_ is known as the microscopic dissociation constant and is the ligand concentration that occupies half of the binding sites.^22^

## Results and Discussion

### Search for EO Orthologs in Plants

To identify EO orthologs in plants, BLAST searches were performed using the amino acid sequences of three previously characterized EO enzymes: FaEO from *Fragaria x ananassa* (Strawberry; accession numbers Q84 V25 and AAO22131),^10^ SlEO from *Solanum lycopersicon* (Tomato; NP_001296292),^7^ and MiEO from *Mangifera indica* (Mango; AFJ53076).^26^ Five putative EO candidates with high sequence similarity to these references were identified in different fruit species: VvEO from *Vitis vinifera* (Grape; XP_003631255), AncEO from *Ananas comosus* (Pineapple; XP_020106159), AccEO from *Actinidia chinensis* (Kiwi; PSR84843), MdEO from *Malus domestica* (Apple; XP_008389642), and MsEO from *Malus sylvestris* (Crab apple; XP_050103939). These sequences were considered EO orthologs for further phylogenetic and functional analyses (Figures 1 and 2; Supplementary Table S3). Some sequences contained additional N-terminal amino acids that were predicted to be signal peptides in VvEO by both SignalP-6.0 (services.healthtech.dtu.dk/services/SignalP-6.0/) and PrediSi (predisi.de/home.html). Therefore, these N-terminal extensions were trimmed back to the conserved MKAW motif, which was present in all EO sequences except FaEO, MsEO, and AncEO (Figure 2). These exceptions either lacked a methionine immediately preceding a lysine (FaEO and MsEO) or encoded an arginine in place of lysine (AncEO) (Figure 2).

**Figure 2:**
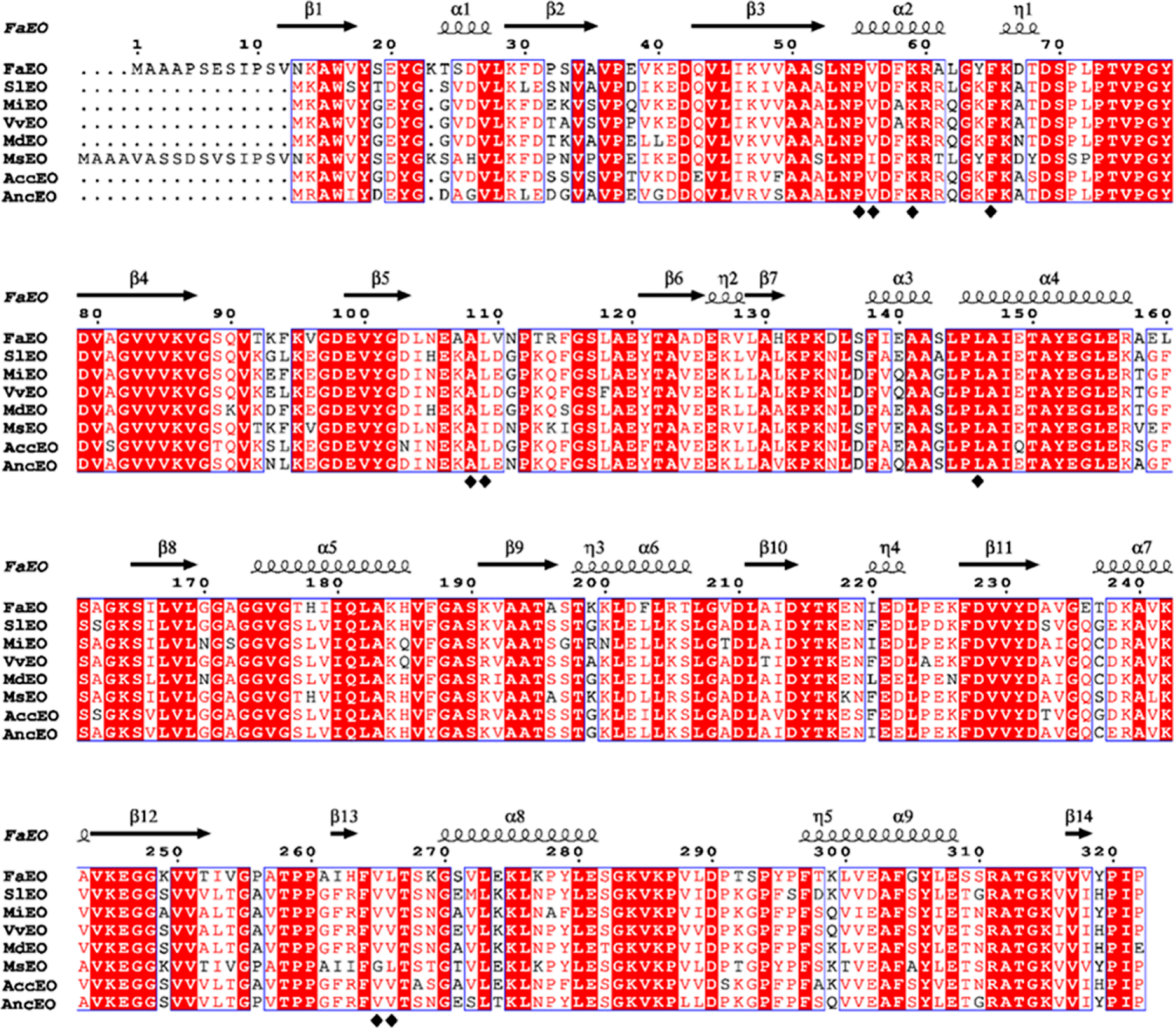
Amino acid sequence alignment of the candidate EOs. The sequences were aligned using the ESPript (espript.ibcp.fr/ESPript/ESPript/).^27^ α-helices are displayed as medium squiggles. β-strands are rendered as arrows, and the η symbol indicates a 3_10_-helix. Red box, white character means strict identity. The amino acids of the acceptor substrate pocket were marked by black diamonds.

The proteins lacking the predicted signal peptides share sequence identities ranging from 67 to 91%, with MsEO exhibiting the highest identity (85%) to FaEO among the candidates (Figure 1A). FaEO, MiEO and SlEO have been demonstrated to catalyze HDMF formation. FaEO shares only moderate amino acid identities (70%) with both MiEO and SlEO.^7,26^ Among the proteins from the Rosaceae family, FaEO clusters more closely with MsEO than with MdEO (Figure 1B). The sequences vary in length from 322 amino acids (FaEO) to 389 amino acids (MdEO), including the predicted signal peptide, and exhibit an almost identical acceptor substrate pocket (Figure 2). This pocket is lined by the side chains of Pro55, Val56, Lys59, Phe65, Ala108, Leu109, Leu146, Val265, and Leu266, which, except for Lys-59, mediate exclusively hydrophobic interactions.^11^

Crystallographic analysis has revealed the molecular mechanism of HDMF biosynthesis by FaEO, which involves reducing the unsaturated exocyclic double bond of the highly reactive precursor HMMF.^11^ FaEO adopts a Rossmann fold (residues 147–264) that forms the NADH-binding site and consists of a six-stranded parallel β-sheet flanked by five α-helices. The substrate-binding domain, composed of two noncontiguous segments (residues 2–146 and 265–321), serves to position the substrate for efficient hydride transfer. Within this domain, Lys59, Val56, and Asn54 are critical for binding HMMF and directing the transfer of the *4R*-hydride from NAD­(P)H to the unsaturated exocyclic C6 carbon of HMMF. A comparison with the FaEO sequence revealed that the seven other EO proteins possess a similar Rossmann fold. The substrate-binding residues Asn54 and Lys59 are highly conserved across all EOs. However, Val56 in FaEO is substituted by an isoleucine in MsEO (Figure 2). Val56 contributes to substrate fixation by anchoring a conserved water molecule via its main chain nitrogen, which in turn forms a hydrogen bond with the substrate. Although both valine and isoleucine are β-branched amino acids, the larger *sec*-butyl side chain of isoleucine is predicted to cause steric perturbation within the binding pocket.

### Expression and Purification of Recombinant EOs from *E. coli*

The corresponding gene sequences of the putative EO proteins were synthesized, cloned, and transformed into *E. coli*. To assess functionality, all eight EO recombinant enzymes, lacking their predicted signal peptides, were expressed in *E. coli* BL21­(DE3), and the proteins were purified and analyzed by SDS-PAGE (Supplementary Figure S1). Catalytic activities of the putative EO enzymes were measured using purified proteins together with the universal surrogate substrate PQ and NADH as cofactor. Enzymatic turnover was monitored by determining the decrease in absorbance at 340 nm, which reflects NADH consumption. Notably, NADH was also depleted in assays containing proteins expressed from the empty vector control, owing to endogenous QR enzyme activity from the host strain.^10^ However, the use of wash buffers containing 30, 40, or 50 mM imidazole during affinity purification of the protein markedly reduced this background activity, effectively minimizing interference from *E. coli*-derived proteins (Supplementary Figure S2).

### Substrate Screening and Kinetic Analysis of Recombinant EOs

Previous study showed that FaEO exhibits regiospecificity, catalyzing the reduction of an *o*-quinone (PQ) but not *p*-quinones.^7,10^ To analyze the specificity of the newly isolated proteins, a substrate screening was performed. Because unstable natural *o*-quinones were not available, we tested a set of stable artificial *o*-quinones (*o*-chloranil, 3,5-di*tert*-butyl-*o*-benzoquinone, 1,2-naphthoquinone, and 4,5-dimethoxy-o-benzoquinone), which are structural analogues to the known active substrate PQ (Figure 3A). Control experiments were performed without the addition of protein fractions. To calculate the *K*_*m*_ and *v*_*max*_ values, we used substrates in concentrations ranging from 0 to 500 μM with an excess of NADH (360 μM). The enzyme activity of the recombinant proteins was calculated by measuring the amount of NADH consumed over a specified period (Figure 3A). NADH kinetics were measured in the presence of an excess concentration of PQ (120 μM) serving as the acceptor substrate.

**Figure 3:**
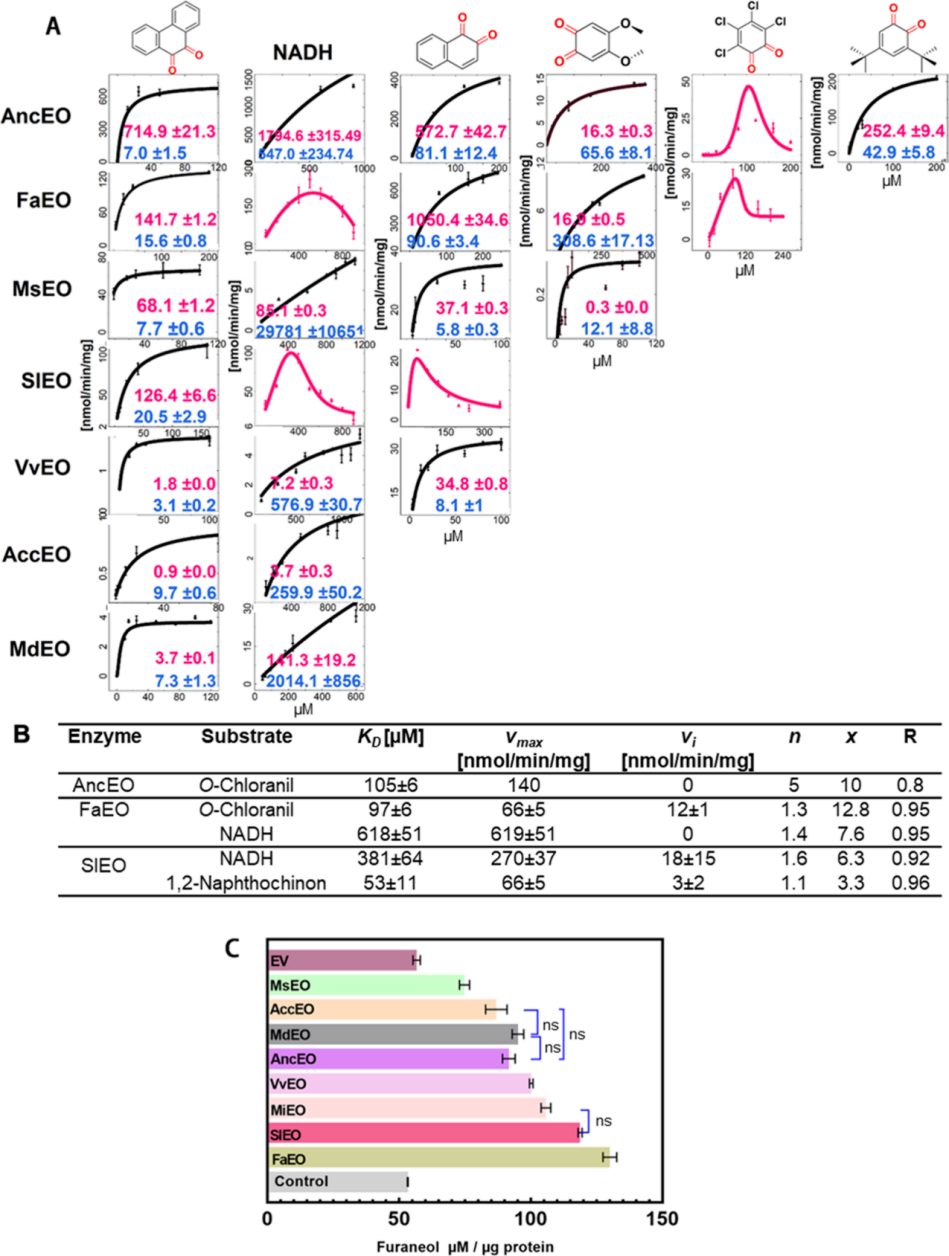
Enzyme activity of recombinant EOs. (A) Reaction rates of EOs as a function of substrate concentration using artificial *o*-quinones and NADH. Rows correspond to individual candidate proteins, and columns indicate the tested substrates: 9,10-phenanthrenequinone, NADH, 1,2-naphthoquinone, 4,5-dimethoxy-*o*-benzoquinone, *o*-chloranil, and 3,5-di*tert*-butyl-*o*-benzoquinone. Michaelis–Menten parameters (*K*_*m*_ in red and *v*_*max*_ in blue) are shown. For reactions exhibiting substrate inhibition, the data were fitted using a recently published equation for asymmetric cooperativity.^22^ (B) Kinetic parameters derived from fitting the asymmetric cooperativity model. (C) HDMF-producing activity of the recombinant EOs. EV, empty vector; control, without protein.

Substrate screening revealed that PQ served as a substrate for all enzymes tested and was reduced following Michaelis–Menten kinetics. Among the EO proteins, AncEO exhibited the highest catalytic activity (*v*_*max*_) toward PQ, while VvEO showed the strongest substrate affinity, reflected by its lowest *K*_*m*_ value. In contrast, assays using quinone analogs demonstrated divergent substrate specificities among the enzymes.

Specifically, AncEO was active toward four candidate *o*-quinones. While the kinetics for three followed the Michaelis–Menten model, *o*-chloranil exhibited pronounced substrate inhibition.

PQ was the preferred substrate for AncEO, as evidenced by its high *v*_*max*_ and low *K*_*m*_. In contrast, FaEO, showed no activity toward 3,5-di*tert*-butyl-*o*-benzoquinone.

Notably, both NADH and *o*-chloranil exhibited unusually strong substrate-inhibition kinetics in the FaEO assays. For MsEO, the active acceptor substrates (PQ, 1,2-naphthoquinone, and 4,5-dimethoxy-*o*-benzoquinone) followed Michaelis–Menten kinetics. Additionally, 1,2-naphthoquinone served as a substrate for both for SlEO and VvEO besides PQ, although its kinetics for SlEO showed significant substrate inhibition.

Michaelis–Menten kinetics is generally applicable to single-substrate enzymes. For bisubstrate enzymes, one substrate is held constant, typically at a saturating concentration, while the other is varied, allowing the reaction to approximate single-substrate behavior. We used a saturating concentration of PQ (120 μM) when determining the kinetics of NADH. However, it was not possible to reach saturating concentrations of NADH because the *Km* values for NADH were extremely high or the reaction displayed substrate-inhibition behavior (Figure 3A). These observations highlight a fundamental limitation in applying classical Michaelis–Menten analysis to this bisubstrate enzyme system.

Data that did not correspond to the Michaelis–Menten model due to substrate inhibition were fitted with eq 2 (Figure 3B). The derivation and interpretation of the equation were published recently.^22^ Under conditions of a fixed NADH concentration (360 μM), the increased *o*-chloranil concentration resulted in the inhibition of both AncEO and FaEO, with the catalytic activity of AncEO being completely inhibited (*v*_*i*_.= 0; Figure 3B). Similarly, increased NADH concentrations inhibited the catalytic activities of FaEO and SlEO, while FaEO’s activity was fully suppressed (*v*_*i*_.= 0; Figure 3B).

Overall, the EOs exhibit distinct substrate profiles and display varying substrate-specific kinetics (Figure 3). Although originally designated as FaQR due to sequence homology with quinone oxidoreductases, FaEO demonstrated a relatively narrow substrate specificity, predominantly reducing PQ among several 1,2- and 1,4-quinones tested.^7,10^ In our experiments, which involved four synthetic *o*-quinones structurally similar to PQ, all eight candidate EOs catalyzed the reduction of PQ and selected other *o*-quinones (Figure 3). Interestingly, AncEO exhibited a broader substrate spectrum, reducing all four artificial *o*-quinones, while MdEO remained specific to PQ. A structural comparison of newly identified FaQR substrates (1,2-naphthoquinone and 4,5-dimethoxy-o-benzoquinone) with inactive *o*-quinones (*o*-cyclohexandione, 2,3-butandione and benzil) suggests that only absolutely planar *o*-quinones can serve as acceptors for FaEO.^7,10^ This is likely due to the planar configuration of the active site (Supplementary Figure S3).^11^ Notably, the MiEO enzyme from Mango catalyzed the reduction of 2-butanone and 3-pentanone, yielding 2-butanol and 3-pentanol, respectively.^26^

Substrate inhibition curves were obtained for five EO-substrate combinations, revealing that excess substrate reduced the reaction rate, similar to the pattern observed for NbUGT72AY1.^22^ These findings suggest that while FaEO can bind NADH and PQ in a random order, the reduction of PQ occurs only when PQ binds first, followed by NADH binding. This mechanism is further supported by the observation that FaEO’s enzymatic activity toward PQ follows Michaelis–Menten kinetics. In contrast, when the concentration of 1,2-naphthoquinone is gradually increased while keeping NADH constant, SlEO exhibits partial substrate inhibition. This suggests that the ternary complex formed when 1,2-naphthoquinone binds first is less catalytically efficient than the alternative complex. A similar inhibition pattern has been observed for AncEO and FaEO with the substrate *o*-chloranil. Although these EOs utilize the same electron donor (NADH), the conformational changes induced by different substrates are not identical, which may explain the differential quinone substrate preferences observed among the EO candidates.

### Recombinant EOs Produce HDMF In Vitro from Fructose-1,6-diphosphate and NADH

FaEO, SlEO, and MiEO produce HDMF when incubated with FDP and NADH overnight, most likely through enzymatic reduction of HMMF, which is chemically generated from the carbohydrate substrate.^7,10,26^ Therefore, the HDMF-forming assay of all purified recombinant EO proteins was quantified by HPLC after incubation with a reaction mixture containing FDP and NADH for 15 h. Control reactions were performed in parallel without recombinant EO proteins as well as proteins isolated from the empty-vector (EV) control. HDMF was detectable in the control samples, indicating a low level of nonenzymatic background formation in the assay system (Figure 3C). All eight recombinant EO proteins, originating from different plant species, were capable of converting FDP into HDMF. Notably, the EOs from apple genotypes (MdEO, MsEO) also produced HDMF, despite the absence of this compound in apple fruits. The concentrations of HDMF generated by the eight EOs were consistently higher than the levels observed in the control. No statistically significant differences were found between several EO pairs, including AccEO and MdEO, AncEO and MdEO, AccEO and AncEO, and MiEO and SlEO. Among the tested enzymes, FaEO showed the highest activity, producing 144% more HDMF than the control, whereas MsEO displayed the lowest activity, generating only 40% above the control level.

Structural analyses have previously shown that Val56, a key active-site residue in FaEO,^11^ is substituted by isoleucine in MsEO (Figure 2). In FaEO, Val56 contributes to substrate fixation by stabilizing a conserved water molecule that forms a hydrogen bond with HMMF (Supplementary Figure S3). In contrast, the bulkier *sec*-butyl side chain of isoleucine is predicted to introduce steric constraints within the substrate-binding pocket. This steric perturbation likely displaces the conserved water molecule from its optimal position, weakening or preventing formation of the hydrogen bond with the substrate. Consequently, substrate stabilization and proper positioning for catalysis are compromised. This structural alteration offers a plausible mechanistic explanation for the markedly reduced HDMF synthesis observed in MsEO relative to FaEO.

### Relationship between EO-Related Gene Expression in Fruit and the Accumulation of HDMF

To evaluate whether *EO* transcripts are present in the candidate fruits, total RNA was extracted from freshly harvested, fully ripe fruits and then reverse-transcribed into cDNA for qPCR analysis. Primers were designed from BLAST-identified *EO* candidate sequences (Supplementary Table S2). Using *FaEO* expression as the reference (set to 1), EO transcripts were detectable in all fruits, including apple (Md) and crab apple (Ms) (Figure 4A). A direct quantitative comparison of transcript levels between fruits is not possible since the reference gene likely exhibits variable expression levels among the different fruit samples, thereby limiting cross-sample normalization.

**Figure 4:**
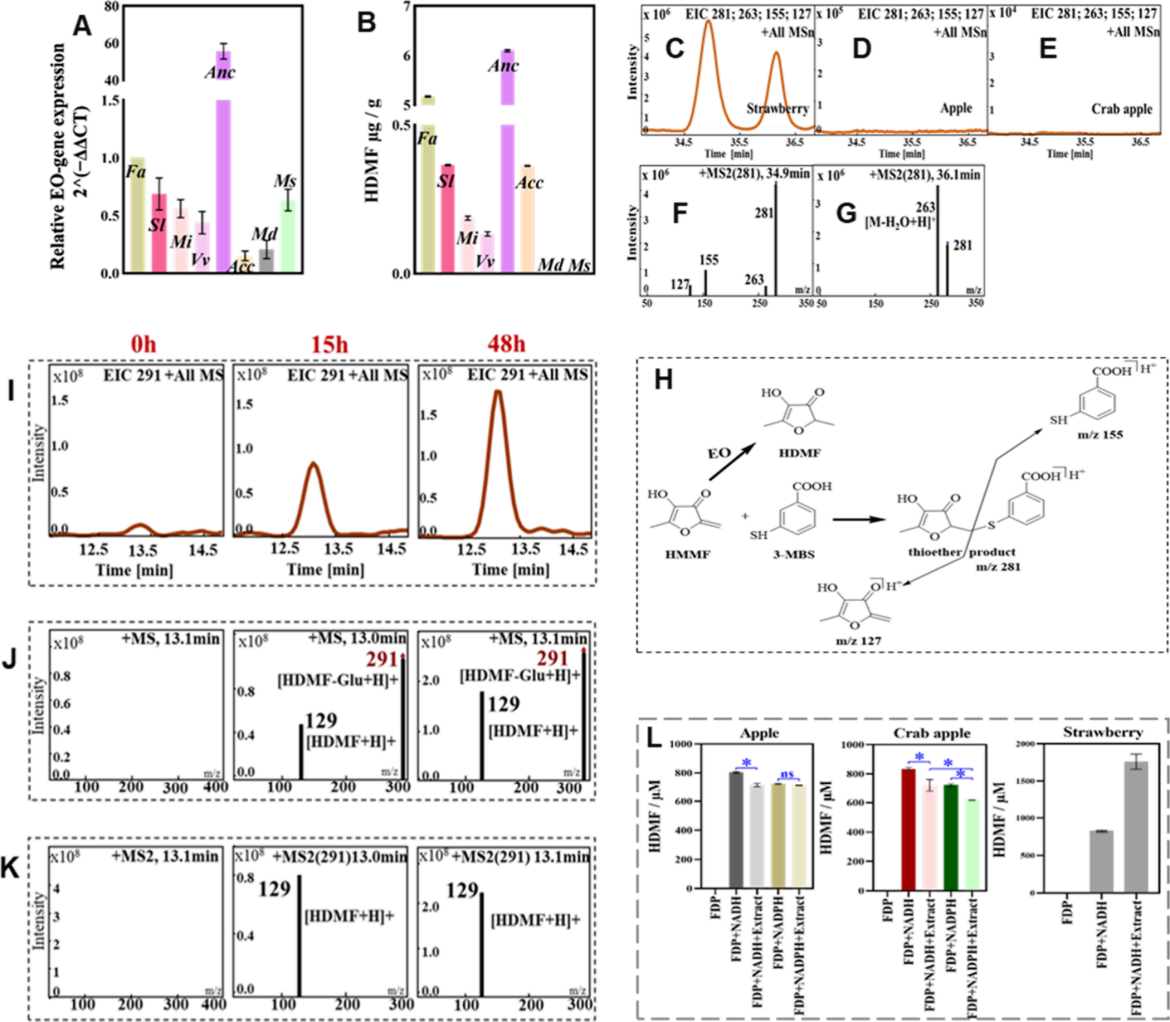
Apples do not produce HDMF. (A) The relative expression levels of *EO* transcripts across the different fruits indicate that *EO* is expressed in all samples analyzed. The *FaEO* gene expression was used as reference (set to 1) and all gene expression was tested with 3 replicates. Due to the varying nature of the fruit materials, a direct comparison of transcript abundance between species is not feasible. (B) HDMF concentration in the corresponding fruits in 100 g fresh fruits. (C) LC-MS analysis of the two isomeric MBS-HMMF thioether products. Fruits were injected with 3-mercaptobenzoic acid (3-MBS) followed by solid-phase extraction on XAD-2. Chromatographic separations of the products isolated from strawberry. (D) MBS-HMMF is not detected in apple. (E) MBS-HMMF is not detected in crab apple. (F) Product ion spectrum (MS2; *m*/*z* 281) of the first peak in panel (C). (G) Product ion spectrum (MS2; *m*/*z* 281) of the second peak in panel (C). (H) Formation and structure of the thioether product as well as their fragments. (I) LC-MS analysis of apple fruit extracts for the detection of HDMF-glucoside. Fruits were injected with HDMF (4 mg/150 g fruit), and glycosides were isolated by solid phase extraction after defined times (0 h: immediate extraction after injection; after 15 h; and 48 h). (J) Mass spectra (MS) of the peak at 13.0 min acquired in the positive mode. (K) Product ion spectra (MS2, *m*/*z* 291) of the peak at 13.0 min acquired in the positive mode. (L) Quantification of HDMF synthesized in control reactions (FDP; FDP+NADH) and by crude protein extracts from the same amount (400g) of fresh apple, crab apple, and strawberry. FDP fructose-1,6-diphosphate.

In parallel with RNA extraction, HDMF was isolated from the same batch of fruit samples (Figure 4B). Qualitative and quantitative analyses were performed using HPLC. Among the eight candidate fruits, all species except apple and crab apple contained detectable levels of HDMF. Pineapple exhibited the highest HDMF concentration (6.1 μg/g fresh fruit), followed by strawberry (5.2 μg/g fresh fruit). HDMF levels in tomato (0.4 μg/g fresh fruit) and kiwi (0.4 μg/g fresh fruit) showed no significant difference, whereas mango (0.19 μg/g fresh fruit) and grape (0.13 μg/g fresh fruit) contained the lowest amounts.

While *EO* transcripts were present in all fruits, including apples, HDMF could not be detected in apple samples. These deviations indicate that EO activity alone does not fully account for HDMF accumulation in fruits.

### In Vivo Studies for the Detection of HMMF in Strawberry Fruit and Apple Fruit

Our previous results demonstrated that apple fruits express a functional *EO* gene, however HDMF is not detectable in apple. To investigate whether the absence of HMMF as a substrate might explain this observation, we attempted to detect HMMF in apple fruit. HMMF has previously been identified as precursor of HDMF in strawberry, tomato, and pineapple following derivatization with 3-mercaptobenzoic acid (3-MBS).^7^ In contrast, it has so far not been detected in raspberry, kiwi, or mango, although these fruits have been shown to contain low concentrations of HDMF.^7,10^

Commercially available 3-MBS rapidly oxidizes to its dimeric form. Therefore, for each experiment, particular care was taken to ensure that quantitative amounts of the 3-MBS monomer were present. The concentration of the 3-MBS monomer was verified daily by LC–MS. The MBS monomer solution was freshly prepared and was injected into strawberry, apple, and crab apple fruits over three consecutive days. After treatment, the fruits were extracted using solid phase extraction, and the resulting extracts were analyzed by LC-MS.

The two isomeric forms of the HMMF-MBS thioether adduct were identified in the strawberry samples based on their retention times (34.9 and 36.1 min) and their MS2 product-ion spectra (MS2), confirming previous findings^7,10^ (Figure 4C). The MS2 of the 34.9 min peak showed characteristic fragments at *m*/*z* 281, 263, 155, and 127, whereas the 36.1 min peak produced fragments at *m*/*z* 281 and 263, confirming the presence of two isomeric HMMF-MBS thioethers (Figure 4F–H). In contrast, the HMMF–MBS adduct was not detectable in apple or crab apple (Figure 4D,E), indicating that HMMF, the natural precursor of HDMF was either absent or present at levels below the detection limit in these fruits.

Isotopic labeling studies have previously established HMMF as a direct biosynthetic precursor to HDMF.^7,10^ This precursor-product relationship was further corroborated through the in vivo trapping of unstable HMMF in strawberry fruit.^7,10^ Injection of 3-MBS into fruits resulted in its reaction with endogenous HMMF, yielding two stable isomeric thioether adducts. This trapping methodology was successfully applied to detect HMMF in other fruits, including pineapple and tomato.^7^ In the present study, the isomeric HMMF-MBS thioether adducts were unequivocally detected in strawberries but were not found in both apple and crab apple tissues. These findings suggest that the lack of HDMF in these *Malus* species can be attributed to the absence of its immediate precursor, HMMF.

### In Vivo Studies on the Metabolism of Injected HDMF in Apple Fruit

Furthermore, we investigated the metabolism of HDMF in apple, as HDMF β-d-glucoside and its malonylated derivative have been reported as downstream metabolites of HDMF in other fruit species. As a control, we first extracted glycosides from untreated apples by SPE but did not detect any HDMF-glucoside and the result was same as the 0 h HDMF injection showed (Figure 4I). To assess whether apples are capable of metabolizing HDMF, we injected fruits with a quantitative amount of HDMF and analyzed the content of its glucoside at 0, 15, and 48 h by LC-MS (Figure 4I–K). HDMF*-β*-d-glucoside appeared in apples 15 and 48 h postinjection, with its concentration increasing over time. Although apples do not biosynthesize HDMF, they are capable of converting the HDMF into its glucoside, most likely via a broad-substrate glycosyltransferase. Therefore, the absence of HDMF-β-d-glucoside in apples is not attributable to a deficiency in glycosylation capacity, but rather to the absence of the HDMF itself.

Plants are equipped with both highly selective and promiscuous glycosyltransferases that catalyze glycosylation of endogenous metabolites while also being preadapted to transform exogenous compounds.^28–31^ These include volatile signaling molecules,^32^ phenolic compounds produced by forest fires,^33^ agrochemicals such as pesticides,^34^ and novel plant metabolites that may arise from random mutations altering the specificity of existing enzymes. This catalytic flexibility enables plants to rapidly detoxify, store, or modulate a wide range of structurally diverse small molecules.^30^

### Protein Extraction for HDMF-Forming from the Fresh Apple and Crab Apple Fruits

HDMF was enzymatically produced in vitro using crude proteins from strawberries in a reaction containing FDP and NADH.^10^ Although a mixture of FDP and NADH alone generated small amounts of HDMF, the yield increased substantially upon addition of strawberry protein extract containing FaEO.^10^ We successfully reproduced these in vitro assays and carried out analogous experiments using fresh apple and crab apple fruits to assess their potential HDMF-forming enzymatic activity (Figure 4L). A reaction containing only FDP served as the negative control, whereas a mixture of FDP and NADH was included to account for the nonenzymatic formation of HDMF in the reaction mixture. Simultaneously, an additional assay was performed in which NADPH was substituted for NADH to explore the cofactor preference of the crude protein extracts. No HDMF was produced in the negative control, whereas approximately 800 μM was formed chemically in the reaction containing only FDP and NADH. In comparison with this nonenzymatic background level, the amount of HDMF synthesized did not significantly alter upon addition of apple crude protein. In fact, the HDMF concentration was significantly reduced when crab apple crude protein was added. HDMF is either degraded by enzymes present in the crude protein extract or, more likely, the substrate FDP is enzymatically cleaved—possibly by fructose-bisphosphate aldolase and fructose-1,6-bisphosphatase, that potentially divert the substrate away from HDMF synthesis.^35,36^

Overall, consistent with previous studies, our experimental results confirm that neither HDMF nor HDMF glycosides were detectable in apples or crab apples,^20^ although qPCR analysis showed expression of functional *MdEO* and *MsEO* genes in both species. Since HMMF could not be trapped as MBS-HMMF in the fruits, and crude protein extracts from fresh apples and crab apples failed to produce HDMF from FDP and NADH, we conclude that HMMF-forming activity is either absent in apple tissue or present at levels below our detection threshold. Furthermore, the downstream enzymes MdEO and MsEO are expressed at low levels in apple fruit and exhibit very weak HDMF-forming activity compared to EOs from other HDMF-producing fruits (Figure 5).

**Figure 5:**
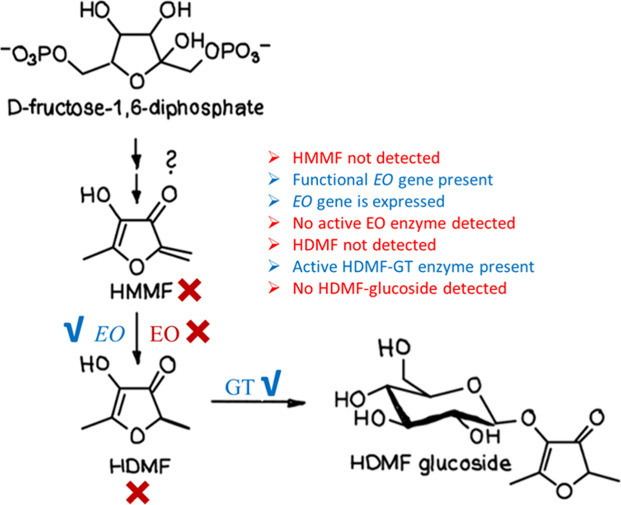
Key findings on HMMF/HDMF pathway in apple fruit. Although apple fruits do not produce HDMF, apple species possess functional downstream genes (*EO* and *GT*) that encode enzymes capable of catalyzing the final steps of HDMF biosynthesis.

The results demonstrate that apples already possess functionally promiscuous downstream genes capable of supporting HDMF biosynthesis, even though the corresponding upstream genes have not yet evolved. This makes the HDMF pathway a compelling example of how new metabolic routes do not arise in a strictly linear manner. Instead, they emerge through the integration of novel reactions into pre-existing metabolic networks, facilitated by the multifunctionality of ancestral enzymes.

Our findings highlight that newly evolving metabolic pathways often originate from secondary or side activities of existing enzymes, which generate new metabolites that subsequently come under selective pressure. If these metabolites confer a fitness advantage to the plant, the associated genes are retained and gradually optimized over evolutionary time.

## Supplementary Material



## Notes

(Table S1) *EO* nucleotide sequences after codon optimization, (Table S2) primer sets used in the study, (Table S3) amino acid sequence of all EO candidates, (Figure S1) SDS-PAGE analysis of recombinant proteins, (Figure S2) enzyme activity from *E. coli* effectively minimized, and (Figure S3) substrate binding to FaEO (PDF)

## Abbreviations and Nomenclature

CoA: coenzyme A
DAD: diode array detector
DMMF: 2,5-dimethyl-4-methoxy-3­(2*H*)-furanone
EDTA: ethylenediaminetetraacetic acid
EIC: extracted ion chromatogram
EO: enone oxidoreductase
FaEO: *Fragaria × ananassa* enone oxidoreductase
FaMAT: *Fragaria × ananassa* malonyltransferase
FaOMT: *Fragaria ananassa* *O*-methyltransferase
FaQR: *Fragaria x ananassa* quinone oxidoreductase
GST: glutathione-S-transferase
HDMF: 4-hydroxy-2,5-dimethyl-3­(2*H*)-furanone
HMMF: 4-hydroxy-5-methyl-2-methylene-3­(2*H*)-furanone
HPLC: high-performance liquid chromatography
IPTG: isopropyl β-d-thiogalactopyranoside
LB: lysogeny broth
LC-MS: liquid chromatography mass spectrometry
PCR: polymerase chain reaction
PMSF: phenylmethylsulfonyl fluoride
PQ: 9,10-phenanthrenequinone
PVPP: polyvinylpolypyrrolidone
SDS PAGE: sodium dodecyl-sulfate polyacrylamide gel electrophoresis
UV: ultraviolet
