# Supplementary material for: Functional Characterization of Enone Oxidoreductase Orthologs Highlights Hidden Furaneol Biosynthetic Capacity in Apples
Source: J Agric Food Chem. 2026 Apr 22;74(17):13898–908. doi: 10.1021/acs.jafc.6c02103 (PMC13154184; doi:10.1021/acs.jafc.6c02103)
Supplement: Supplementary file 1 [file jf6c02103_si_001.pdf]

Supplementary information

Functional Characterization of Enone Oxidoreductase Orthologs Highlights Hidden Furaneol Biosynthetic Capacity in Apples

Chan Li, Katharina Jahr, Yujun Sun, Benedikt Johannes Thalhammer, Julia Schweppe, Xiran Wang, Timothy D. Hoffmann, Thomas Hoffmann, Wilfried Schwab*

Biotechnology of Natural Products, School of Life Sciences, Technical University of Munich, 85354 Freising, Germany

Corresponding Author's email address: wilfried.schwab@tum.de

Table S1. The *EO* nucleotide sequences after codon optimization.

	Complete *EO* nucleotide sequences after codon optimization
***Mangifera indica* (*MiEO*)**	atgaaagcgtgggtgtatggagaatatggaggagtcgatgtattgaaatttgatgagaaagtttctgtgcctcaagtgaaggaagatcaggtgttgatcaaggttgttgctgcggctcttaatcctgttgatgctaagcgaagacagggcaagttcaaggccaccgattctcccctcccgactgttccagggtatgatgtagctggtgtggtggtcaaggttggcagtcaagtgaaggaatttaaggagggagatgaggtatatggggacataaatgagaaggcactggaagggccgaaacaatttggctccttagccgagtacaccgcagttgaagaaaagttattggcactgaaaccaaaaaatttggattttgttcaggctgctggccttcctcttgctattgagacggcctatgagggtctagaaagaactgggttctctgctggtaaatctattcttgttttgaatggttctggtggtgttggaagcttggtgattcagctagcaaaacaagtatttggtgcttcaaaagttgccgcaacttcaggaaccagaaacttggaacttttgaagagcttgggtactgatttggctattgactacaccaaagaaaatattgaagatttgccagaaaagtttgatgtggtctatgatgctattgggcaatgtgatcgagcagtaaaggtagtgaaagaaggggggtgctgtggtggccctaacaggtgctgtaacacctccaggcttcagatttgtagttacttcaaatggagctgttctgaaaaaattaaatgcatttctagagagtggaaaggtaaagcctgtaatagaccccaaggggccatttcctttcagccaggttattgaggctttctcttacattgaaacgaacagagctactggaaaggtggttatatacccaattcct
***Malus domestica* (*MdEO*)**	atgaaggcgtgggtttatggggaatacgggggtgttgatgttttgaagttcgacaccaaagttgcggttcctgagttgctggaggaccaggttctggttaaggttgtcgctgcggctctcaacccggttgacttcaagaggaggcagggcaagttcaagaacactgattctcctcttccgactgttccaggctatgatgttgccggcgtggtggtgaaggttggcagtaaagtgaaggactttaaagagggggatgaagtgtatggagacatacatgagaaagctttggaagggcctaaacaatctggctctcttgcagagtacactgccgttgaagagaggttactggcagcaaagcccaagaatttggattttgctgaggctgcttcacttcctcttgcaatcgagacagcttatgagggtcttgaaaagaccgggttttctgccggtaaatctttgcttgttctgaatggtgctggtggagttggaagcctagtgattcagctagcaaaacatgtatttggagcttcacgaatagcagccacttcaagcactggaaagctggagttattgaagagcttgggtgctgatttagcgatcgactacactaaggagaacttggaagaattgccggaaaattttgatgtcgtctatgatgctattggtcaatgtgacaaagcagtgaaggtggtgaaagaaggggggcagtgtggtggccttaacaggtgcagtaacacctccgggtttcagattcgtggttacttccaacggagcagttctgaagaaactaaacccgtacttagagactgggaaggtgaagcctgtgatagaccccaaagggcccttccccttctccaagcttgtggaggctttctcttaccttgaaacaaatcgggccaccggaaaggtagtcatacatccaattgaa
***Malus sylvestris* (MsEO)**	atggcggctgctgtagcttcaagtgattcagtttccataccatctgtaaacaaggcgtgggtgtactcagagtacggaaagtctgctcatgttttgaagttcgatccgaatgttcctgttcctgaaataaaggaagaccaggtgctgatcaaggtggttgccgcatctctcaacccaattgatttcaaaaggacgcttggctacttcaaggactatgactcttcacccccctacagttccagggtatgatgttgctggtgtggtggtgaaagtaggaagccaagtgacaaagtttaaggtgggggatgaggtatatggggatctcaatgagaaggctattgataacccaaaaaagatcggatctttggccgagtacactgctgcagaagaaagagtgttggctctcaagccaaaaaatttgagttttgttgaagctgctagccttcccctggccattgagaccgcctatgaagggctcgaacgagttgaattctctgccggtaaatccatccttgttttgggaggcgctgggggagtcggaacacatgttattcagctagcaaggcatgtgtttggtgcttcaaaagtagcagctacagcgagcacgaaaaaactcgatcttttgagaagcttgggtgctgatttggctattgattacaccaagaagaacttcgaagacctgcccgagaaatttgatgtggtttatgatgcagttgggcagagtgacagggcattgaaggcggtgaaggaaggcgggaaggttgtgacaatcgtaggtccagcaacgccaccggcaatcatatttggactgacttcaaccggaactgtattagagaaactgaagccttacttggagagtgggaaggtgaagccggtgcttgatcccacaggcccgtatccattttcgaagactgttgaagcatttgcctatcttgaaacctccagagctaccggaaaggtggttgtgtatcccatccca
***Actinidia chinensis* (*AccEO*)**	atgaaggcttgggtctacggagattatggaggtgttgatgttttgaagtttgattccagtgtttcggtgccaacagtgaaggatgatgaggtcttgatcagggtttttgcggctgctctcaaccctgttgatttcaagaggaggcagggaaagttcaaggcctccgactcgcctcttccgacagttccaggttacgatgtttccggtgtggtggttaaagtaggcacccaagtcaagagcctgaaggaaggggacgaagtgtacggtaacattaacgagaaagccttagacgggcctaaacagttcggctctttggccgagttcactgctgttgaggagaagttattggctctcaaacctaagaatctcgattttgctgaggccgcgggcctgccgctcgcgattcagactgcctacgaaggcctcgaacggtctgggttttcatccggaaaatctgtccttgttttgggggggtgccggtggagtcggatcgctagtaattcagctggcaaaacatgtctttggggcttcaagagttgcagcaacttccagcactggaaagttagaaatactcaaaagcttgggtgctgatttggctgtagactacaccaaggaaagcttcgaagacctacccgaaaaatttgacgtggtctatgacactgtcggcagggcgataaggcggtgaagggtggtgaaggaaggcggaagtgtggtggtgctaaccggtgcagtcacaccacccggtttcagatttgtagtcactgccagtggagcagtcttggaaaaattgaatccgtttttggaaagcgggaaggtgaagccggtggtcgattccaaggggccatttccatttgccaaggttgttgaagccttttcttacgttgaaacgaaccgagctactggaaaagtagttatacatccaattcca
***Ananas comosus* (*AncEO*)**	atgagggcgtggatctacgacgagtacggggacgcgggggttttgcgcctcgaggacggggtcgccgttcccgaggtcggggacgatcaggtcctcgttagggtttccgccgccgcgctcaacccccgtcgatttcaagcggaggcagggcaaatttaaggccacggattcgcccttacctacggtaccgggttacgatgtggccggcgtcgtggtgaaggttggtagccaagtcaagaatctaaaagaaggggacgaagtatacggggacataaacgagaaggcgttggagaatccgaagcaattcggatcgcttgcggagtacactgcagtcgaggagaagctgctggctgtaaaacctaagaatctggactttgcgcaggctgctagtcttcctttggccatcgagacggcatatgaaggccttgaaaaagctggattttccgcgggtaaatcagttcttgttttgggtggtgctggcggagtcggctcgctcgtgattcagctagcaaagcatgtctacggtgcgtcgaaagttgcggccacttctagcacaggaaagctggagctgttgaagagcctgggtgccgacttagcgatcgactacaccaaggaaaacattgaagaactacctgagaagtttgatgtagtgtatgatgctgttggtcagtgcgaaagagcggtgaaagcagtgaaagaagggggcagcgtcgtggtgctcacgggccctgtcacgcccccgggcttcagattcgtcgtcacgtccaacggggagagcctgacgaagctcaacccttatctcgaaagcgggaaggtgaagcctcttctggacccgaaggggccattccctttctctcaggtggttgaggcattctcttatctcgaaaccggaagagctacgggaaaggtcgttatctatccaattcct
***Vitis vinifera* (*VvEO*)**	atgaaggcttgggtttatggagactatggaggagttgatgttttgaaatttgatacagctgtttcggtgcctccagtgaaggaggaccaggttctgatcaaggttgttgctgcagcgcttaaccctgttgatgccaagaggaggcaggggaaattcaaggccactgattctcctcttccgactgttcctggatatgatgtggctggggtggtggtaaaggttggaagccaagtaaaggaactgaaggaaggggatgaagtatatggagacataaacgagaaagcattggatgggcctaagcagtttggctcgtttgcagaatacactgctgttgaagagaaactgttggctctgaaaccaaagaatctggattttgttcaagctgctggacttcctcttgcaattgagacagcctatgagggtctcgaaaggactggattttctgctggtaaatctattcttgttttgggtggggctggtggagttggaagccttgtcatccagcttgcgaaacaagtgttcggtgcctcaagagttgccgctacatcaagcacagcgaaactggagctcttgaagagcttgggagctgatttgacgattgactacactaaggagaactttgaagatttggcagagaaattcgatgtagtttatgatgcagttgggcagtgtgataaggcagtgaaggtagtgaaagaaggaggcagcgtggtggcattaacaggtgccgtgacgcctcctggtttcagatttgtagtaacttcaaacggagaggtgttgaagaaactgaacccatatttggagagcgggaaggtgaagccagtggttgatcccaagggaccatttccattttctcaggttgttgaagctttctcctacgttgaaacaagccgagccactggaaagatagttatacatccgattcca
***Fragaria x ananassa* (*FaEO*)**	atggctgcagctccaagtgagtccataccctctgtaaataaggcctgggtctattcagagtatggaaaaacttctgatgttctcaagtttgatccaagtgtggctgttcctgaaattaaagaggatcaggtgctgatcaaggttgttgctgcttctcttaacccagttgattttaagagggctcttggttacttcaaggacactgactctcccctacctacaattccagggtatgatgtagctggtgtggtggtaaaggtaggaagccaagtaaccaagtttaaggtgggggatgaagtgtatggggatctcaatgagacagcattggtgaacccaccaaggtttgggtctttggcagagtacactgctgcagatgaaagagtattggctcacaaacccaaaaacctgagctttattgaagctgctagccttcccttggctattgaaactgcccatgaagggcttgaaagagctgaactttctgctggtaaatccgtccttgttttgggaggcgctgggggtgttggaacacatattattcagcttgcaaagcatgttttttggtgcttccaaagtagcagctactgcaagcactaagaaactggatttgttgagaaccttgggtgctgatttggctatcgattacaccaaggagaacttcgaggacctgccagagaaatttgatgtagtgtatgatgcagttggggagacagataaggctgtgaaggcggtgaaagaaggcgggaaggttgtaacaatagtaggtccagcaacgccaccggctatcctttttgtgcttacctctaaagggtctgtgttggagaaactgaagccttacttggagagtgggaaggtgaagccagttcttgatcccacaagtccatatccctttactaaagttgttgaagcatttggttaccttgagagttccagagctaccggaaaggtggttgtgtatcccatc
***Solanum lycopersicum* (*SiEO*)**	atggaagctctgttatcttccactactcttcaactcaaaccccttcatccaccctcttctttctcttctcttcactctccattttcttctatttctgttcttagagtaaagggtagcaaaaaagctgaaacttttattcaaagaagcaatttttccactgttttgccacttagagtctctgctagttctcaagcagcagctgctgaaacatccacaagcatttctattccttctgaaatgaaagcttggagttatactgattatggaagtgttgatgttttgaagttggagtccaatgttgcagttcctgacattaaggaagatcaagtcttgattaagattgttgctgctgctcttaaccctgttgattttaaacgacggctcggaaaatttaaggccactgattctccacttccaactgtgccaggttatgatgttgctggtgtggtagtgaaagttggtagtcaggtaaagggattaaaagaaggggatgaagtatatggagatatacatgagaaagcattagatggaccaaagcaatttggatccttagctgagtacactgctgttgaagagaaactagttgctttgaaacccaagaatcttagttttgcggaggctgctgctcttcctctagctattgagactgcttatgaaggtcttgagaaagctggattttcttctggcaaatccattcttgttttgggaggtgctggcggtgttggatccctagtaatccagcttgctaaacatgtatttggtgcttctaaagtagcagctacctctagcacagggaagttggaactcctgaaaagcttaggagctgatttagctattgactacactaaggagaattttgaagatctgcctgacaagtttgatgttgtttatgattcagttggtcagggtgaaaaggcggtgaaggtagtgaaggaaggtgggagtgtggtagtgctaacaggggcagtaactccaccaggttttagatttgtggtgacatcaaatggagagatgttgaagaaactgaacccatacttggaaagtgggaaggtgaaaccagtgatagatccaaagggaccattctcatttgataaggttgtggatgctttctcttatcttgaaactggaagagccactgggaaagttgttattcatcccattcct

Table S2. Primer sets used in the study.

	Primer for EO gene
	**Primers(5' - 3')(Forward/Reverse)**
Actinidia chinensis	F: CCCTGTTTTGCCCCTGAGAA
	R: ATCTCCGTAGACCCAAGCCT
Fragaria ananassa	F: AGCGAGTCCATACCCTCTGT
	R: ACCGGGTTAAGAGAAGCAGC
Vitis vinifera	F: CCTTGTCATCCAGCTTGCGA
	R: ACTGCCTTATCACACTGCCC
Mangifera indica	F: GATCAAGGTTGTTGCTGCGG
	R: ATACCCTGGAACAGTCGGGA
Malus domestica	F: TGTCGTCTATGATGCTATTGGTCA
	R: TCCCAGTCTCTAAGTACGGGT
Malus sylvestris	F: CAGGTGCTGATCAAGGTGGT
	R: TACCCTGGAACTGTAGGGGG
Ananas comosus	F: ATGATGCTGTTGGTCAGTGC
	R: GGACGTGACGACGAATCTG
Solanum lycopersicum	F: CTTAGTTTTGCGGAGGCTGC
	R: CCCTGTGCTAGAGGTAGCTG

	Primer for Housekeeping gene	Gene accession Number
Actinidia chinensis	F: CTGTGAAACTGCGAATGGCTC	AF419792
	R: TTCCAGAAGTCGGGGTTTGT	
	F: GCAGGAATCCATGAGACTACC	EF063572
	R: GTCTGCGATACCAGGGAACAT	
Fragaria ananassa	F: TTGGCAGCGGGACTTTACC	EU727547
	R: CGGTTGTGTGACGCTGTCAT	
	F: GTGGCGTCAAGCGTATCTCC	AB197150.1
	R: TGTCCTTCCCTGCCTCTTGA	
Vitis vinifera	F: TTCCTCCTTCGCCTCCGATT	XM_002282480.5
	R: CACCCGCAAATCCAGCCTTC	
	F: CAGCACCTTCCAGCAGATGT	XM_002282480.5
	R: CGAAAAGCCAACTAAAGGGGG	
Mangifera indica	F: GTCGCTACCTCACTGCTTCA	OP047694
	R: CACAGACACTGGACTTGACATTA	
Malus domestica	F: GGATTTACTGTGTTGGTGAAG	AT3G49660.1
	R: TGCCAATTACCTCCTTTTCGTG	
Malus sylvestris	F: TGTGGAAGGAGATCAAAGCGCA	MDP0000229861
	R: CGCGTTGCTTCTTTGCTGCA	
Solanum lycopersicum	F: TTTGCATTCCCTGACTGTTTGC	NM_001330119.1
	R: CTGCGAACCCAGCCTTGA	
Ananas comosus	F: AACATTGTTCGTTGGGAAATGGC	XM_020227265.1
	R: CTAGGAAATACAGCCCTCGGC	

Table S3. The amino acid sequence of all EO candidates.

	complete sequence	without signal pepitide
***Mangifera indica* (*Mi*EO)**	>AFJ53076.1 enone oxidoreductase [Mangifera indica]<br>METILASAPSKFTSFHSFSQRFSLAFRENRRKNVVNVNGASSSYLPLIVR<br>ANSQAAPASTEATKVSSVPSEMKAWVYGEYGGVDVLKFDEKVSVPQV<br>KEDQVLIKVVAAALNPVDAKRRQGKFKATDSPLPTVPGYDVAGVVVKV<br>GSQVKEFKEGDEVYGDINEKALEGPKQFGSLAEYTAVEEKLLALKPKNL<br>DFVQAAGLPLAIETAYEGLERTGFSAGKSILVLNGSGGVGSLVIQLAKQV<br>FGASKVAATSGTRNLELLKSLGTDLAIDYTKENIEDLPEKFDVVYDAIGQ<br>CDRAVKVVKEGGAVVALTGAVTPPGFRFVVTSNGAVLKKLNAFLESGK<br>VKPVIDPKGPFPFSQVIEAFSYIETNRATGKVVIYPIP	MKAWVYGEYGGVDVLKFDEKVSVPQVKEDQVLIKVVAAALNPVD<br>AKRRQGKFKATDSPLPTVPGYDVAGVVVKVGSQVKEFKEGDEV<br>YGDINEKALEGPKQFGSLAEYTAVEEKLLALKPKNLDFVQAAGLP<br>LAIETAYEGLERTGFSAGKSILVLNGSGGVGSLVIQLAKQVFGASK<br>VAATSGTRNLELLKSLGTDLAIDYTKENIEDLPEKFDVVYDAIGQC<br>DRAVKVVKEGGAVVALTGAVTPPGFRFVVTSNGAVLKKLNAFLE<br>SGKVKPVIDPKGPFPFSQVIEAFSYIETNRATGKVVIYPIP
***Malus domestica* (*Md*EO)**	>XP_008389642.1 2-methylene-furan-3-one reductase-like [Malus domestica]<br>MLTTTAALTSTASQLTSPNQISPRFSFTFRENNNRIAVPKSSTTQRSKLS<br>VGFPALRVSASSRSAPASAEASKVTVLPSEMKAWVYGEYGGVDVLKFD<br>TKVAVPELLEDQVLVKVVAAALNPVDFKRRQGKFKNTDSPLPTVPGYDV<br>AGVVVKVGSKVKDFKEGDEVYGDIHEKALEGPKQSGSLAEYTAVEERLL<br>AAKPKNLDFAEAASLPLAIETAYEGLEKTGFSAGKSLLVLNGAGGVGSLV<br>IQLAKHVFGASRIAATSSTGKLELLKSLGADLAIDYTKENLEELPENFDVV<br>YDAIGQCDKAVKVVKEGGSVVALTGAVTPPGFRFVVTSNGAVLKKLNPY<br>LETGKVKPVIDPKGPFPFSKLVEAFSYLETNRATGKVVIHPIE	MKAWVYGEYGGVDVLKFDTKVAVPELLEDQVLVKVVAAALNPVD<br>FKRRQGKFKNTDSPLPTVPGYDVAGVVVKVGSKVKDFKEGDEV<br>YGDIHEKALEGPKQSGSLAEYTAVEERLLAAKPKNLDFAEAASLP<br>LAIETAYEGLEKTGFSAGKSLLVLNGAGGVGSLVIQLAKHVFGAS<br>RIAATSSTGKLELLKSLGADLAIDYTKENLEELPENFDVVYDAIGQ<br>CDKAVKVVKEGGSVVALTGAVTPPGFRFVVTSNGAVLKKLNPYL<br>ETGKVKPVIDPKGPFPFSKLVEAFSYLETNRATGKVVIHPIE
***Malus sylvestris* (*Ms*EO)**	>XP_050103939.1 2-methylene-furan-3-one reductase [Malus sylvestris]<br>MAAAVASSDSVSIPSVNKAWVYSEYGKSAHVLKFDPNVPVPEIKEDQVL<br>IKVVAASLNPIDFKRTLGYFKDYDSSPPTVPGYDVAGVVVKVGSQVTKF<br>KVGDEVYGDLNEKAIDNPKKIGSLAEYTAAEERVLALKPKNLSFVEAASL<br>PLAIETAYEGLERVEFSAGKSILVLGGAGGVGTHVIQLARHVFGASKVAA<br>TASTKKLDLLRSLGADLAIDYTKKNFEDLPEKFDVVYDAVGQSDRALKAV<br>KEGGKVVTIVGPATPPAIIFGLTSTGTVLEKLKPYLESGKVKPVLDPTGPY<br>PFSKTVEAFAYLETSRATGKVVVYPIP	MAAAVASSDSVSIPSVNKAWVYSEYGKSAHVLKFDPNVPVPEIKE<br>DQVLIKVVAASLNPIDFKRTLGYFKDYDSSPPTVPGYDVAGVVVK<br>VGSQVTKFKVGDEVYGDLNEKAIDNPKKIGSLAEYTAAEERVLAL<br>KPKNLSFVEAASLPLAIETAYEGLERVEFSAGKSILVLGGAGGVGT<br>HVIQLARHVFGASKVAATASTKKLDLLRSLGADLAIDYTKKNFEDL<br>PEKFDVVYDAVGQSDRALKAVKEGGKVVTIVGPATPPAIIFGLTST<br>GTVLEKLKPYLESGKVKPVLDPTGPYPFSKTVEAFAYLETSRATG<br>KVVVYPIP
***Actinidia chinensis* (*Acc*EO)**	>PSR84843.1 2-methylene-furan-3-one reductase [Actinidia chinensis var. chinensis]<br>MEAMLSSTSLQLRPLPPHSLSPLSLSRLQLPRGRTPINCFQKTSQSPVL<br>PLRIYASSQTAPASTAAATSSSIPSQMKAWVYGDYGGVDVLKFDSSVSV<br>PTVKDDEVLIRVFAAALNPVDFKRRQGKFKASDSPLPTVPGYDVSGVVV<br>KVGTQVKSLKEGDEVYGNINEKALDGPKQFGSLAEFTAVEEKLLALKPK<br>NLDFAEAAGLPLAIQTAYEGLERSGFSSGKSVLVLGGAGGVGSLVIQLA<br>KHVFGASRVAATSSTGKLEILKSLGADLAVDYTKESFEDLPEKFDVVYDT<br>VGQGDKAVKVVKEGGSVVVLTGAVTPPGFRFVVTASGAVLEKLNPFLE<br>SGKVKPVVDSKGPFPFAKVVEAFSYVETNRATGKVVIHPIP	MKAWVYGDYGGVDVLKFDSSVSVPTVKDDEVLIRVFAAALNPVD<br>FKRRQGKFKASDSPLPTVPGYDVSGVVVKVGTQVKSLKEGDEVY<br>GNINEKALDGPKQFGSLAEFTAVEEKLLALKPKNLDFAEAAGLPL<br>AIQTAYEGLERSGFSSGKSVLVLGGAGGVGSLVIQLAKHVFGAS<br>RVAATSSTGKLEILKSLGADLAVDYTKESFEDLPEKFDVVYDTVG<br>QGDKAVKVVKEGGSVVVLTGAVTPPGFRFVVTASGAVLEKLNPF<br>LESGKVKPVVDSKGPFPFAKVVEAFSYVETNRATGKVVIHPIP
***Ananas comosus* (*Anc*EO)**	>XP_020106159.1 2-methylene-furan-3-one reductase-like [Ananas comosus]<br>MLCTLHCPSSSSLSLLPLPSPSKPYLAPPLLRPRSLRRGGGGVGGGGG<br>GVVGARYKLGGAYPSTRVSASSKPGPASADAVVAAPSEVPEQMRAWIY<br>DEYGDAGVLRLEDGVAVPEVGDDQVLVRVSAAALNPVDFKRRQGKFK<br>ATDSPLPTVPGYDVAGVVVKVGSQVKNLKEGDEVYGDINEKALENPKQ<br>FGSLAEYTAVEEKLLAVKPKNLDFAQAASLPLAIETAYEGLEKAGFSAGK<br>SVLVLGGAGGVGSLVIQLAKHVYGASKVAATSSTGKLELLKSLGADLAID<br>YTKENIEELPEKFDVVYDAVGQCERAVKAVKEGGSVVVLTGPVTPPGFR<br>FVVTSNGESLTKLNPYLESGKVKPLLDPKGPFPFSQVVEAFSYLETGRA<br>TGKVVIYPIP	MRAWIYDEYGDAGVLRLEDGVAVPEVGDDQVLVRVSAAALNPV<br>DFKRRQGKFKATDSPLPTVPGYDVAGVVVKVGSQVKNLKEGDE<br>VYGDINEKALENPKQFGSLAEYTAVEEKLLAVKPKNLDFAQAASL<br>PLAIETAYEGLEKAGFSAGKSVLVLGGAGGVGSLVIQLAKHVYGA<br>SKVAATSSTGKLELLKSLGADLAIDYTKENIEELPEKFDVVYDAVG<br>QCERAVKAVKEGGSVVVLTGPVTPPGFRFVVTSNGESLTKLNPY<br>LESGKVKPLLDPKGPFPFSQVVEAFSYLETGRATGKVVIYPIP
***Vitis vinifera* (*Vv*EO)**	>XP_003631255.1 2-methylene-furan-3-one reductase [Vitis vinifera]<br>MEAVLNSTTTYLPTPRHLSSTHSYPLPFSLSFRESRRKFSPKQASVLRV<br>FASSQAAPASSAAVTIPSQMKAWVYGDYGGVDVLKFDTAVSVPPVKED<br>QVLIKVVAAALNPVDAKRRQGKFKATDSPLPTVPGYDVAGVVVKVGSQ<br>VKELKEGDEVYGDINEKALDGPKQFGSFAEYTAVEEKLLALKPKNLDFV<br>QAAGLPLAIETAYEGLERTGFSAGKSILVLGGAGGVGSLVIQLAKQVFGA<br>SRVAATSSTAKLELLKSLGADLTIDYTKENFEDLAEKFDVVYDAVGQCDK<br>AVKVVKEGGSVVALTGAVTPPGFRFVVTSNGEVLKKLNPYLESGKVKPV<br>VDPKGPFPFSQVVEAFSYVETSRATGKIVIHPIP	MKAWVYGDYGGVDVLKFDTAVSVPPVKEDQVLIKVVAAALNPVD<br>AKRRQGKFKATDSPLPTVPGYDVAGVVVKVGSQVKELKEGDEV<br>YGDINEKALDGPKQFGSFAEYTAVEEKLLALKPKNLDFVQAAGLP<br>LAIETAYEGLERTGFSAGKSILVLGGAGGVGSLVIQLAKQVFGAS<br>RVAATSSTAKLELLKSLGADLTIDYTKENFEDLAEKFDVVYDAVG<br>QCDKAVKVVKEGGSVVALTGAVTPPGFRFVVTSNGEVLKKLNPY<br>LESGKVKPVVDPKGPFPFSQVVEAFSYVETSRATGKIVIHPIP
***Fragaria ananassa* (*Fa*EO)**	>AAO22131.1 identical to Q84V25.1 quinone oxidoreductase [Fragaria x ananassa]<br>MAAAPSESIPSVNKAWVYSEYGKTSDVLKFDPSVAVPEVKEDQVLIKVV<br>AASLNPVDFKRALGYFKDTDSPLPTVPGYDVAGVVVKVGSQVTKFKVG<br>DEVYGDLNEAALVNPTRFGSLAEYTAADERVLAHKPKDLSFIEAASLPLA<br>IETAYEGLERAELSAGKSILVLGGAGGVGTHIIQLAKHVFGASKVAATAS<br>TKKLDFLRTLGVDLAIDYTKENIEDLPEKFDVVYDAVGETDKAVKAVKEG<br>GKVVTIVGPATPPAIHFVLTSKGSVLEKLKPYLESGKVKPVLDPTSPYPF<br>TKLVEAFGYLESSRATGKVVVYPIP	MAAAPSESIPSVNKAWVYSEYGKTSDVLKFDPSVAVPEVKEDQV<br>LIKVVAASLNPVDFKRALGYFKDTDSPLPTVPGYDVAGVVVKVGS<br>QVTKFKVGDEVYGDLNEAALVNPTRFGSLAEYTAADERVLAHKP<br>KDLSFIEAASLPLAIETAYEGLERAELSAGKSILVLGGAGGVGTHII<br>QLAKHVFGASKVAATASTKKLDFLRTLGVDLAIDYTKENIEDLPEK<br>FDVVYDAVGETDKAVKAVKEGGKVVTIVGPATPPAIHFVLTSKGS<br>VLEKLKPYLESGKVKPVLDPTSPYPFTKLVEAFGYLESSRATGKV<br>VVYPIP
***Solanum lycopersicum* (*Sl*EO)**	>NP_001296292.1 2-methylene-furan-3-one reductase [Solanum lycopersicum]<br>MEALLSSTTLQLKPLHPPSSFSSLHSPFSSISVLRVKGSKKAETFIQRSN<br>FSTVLPLRVSASSQAAAAETSTSISIPSEMKAWSYTDYGSVDVLKLESNV<br>AVPDIKEDQVLIKIVAAALNPVDFKRRLGKFKATDSPLPTVPGYDVAGVV<br>VKVGSQVKGLKEGDEVYGDIHEKALDGPKQFGSLAEYTAVEEKLVALKP<br>KNLSFAEAAALPLAIETAYEGLEKAGFSSGKSILVLGGAGGVGSLVIQLA<br>KHVFGASKVAATSSTGKLELLKSLGADLAIDYTKENFEDLPDKFDVVYDS<br>VGQGEKAVKVVKEGGSVVVLTGAVTPPGFRFVVTSNGEMLKKLNPYLE<br>SGKVKPVIDPKGPFSFDKVVDAFSYLETGRATGKVVIHPIP	MKAWSYTDYGSVDVLKLESNVAVPDIKEDQVLIKIVAAALNPVDF<br>KRRLGKFKATDSPLPTVPGYDVAGVVVKVGSQVKGLKEGDEVY<br>GDIHEKALDGPKQFGSLAEYTAVEEKLVALKPKNLSFAEAAALPL<br>AIETAYEGLEKAGFSSGKSILVLGGAGGVGSLVIQLAKHVFGASK<br>VAATSSTGKLELLKSLGADLAIDYTKENFEDLPDKFDVVYDSVGQ<br>GEKAVKVVKEGGSVVVLTGAVTPPGFRFVVTSNGEMLKKLNPYL<br>ESGKVKPVIDPKGPFSFDKVVDAFSYLETGRATGKVVIHPIP

A

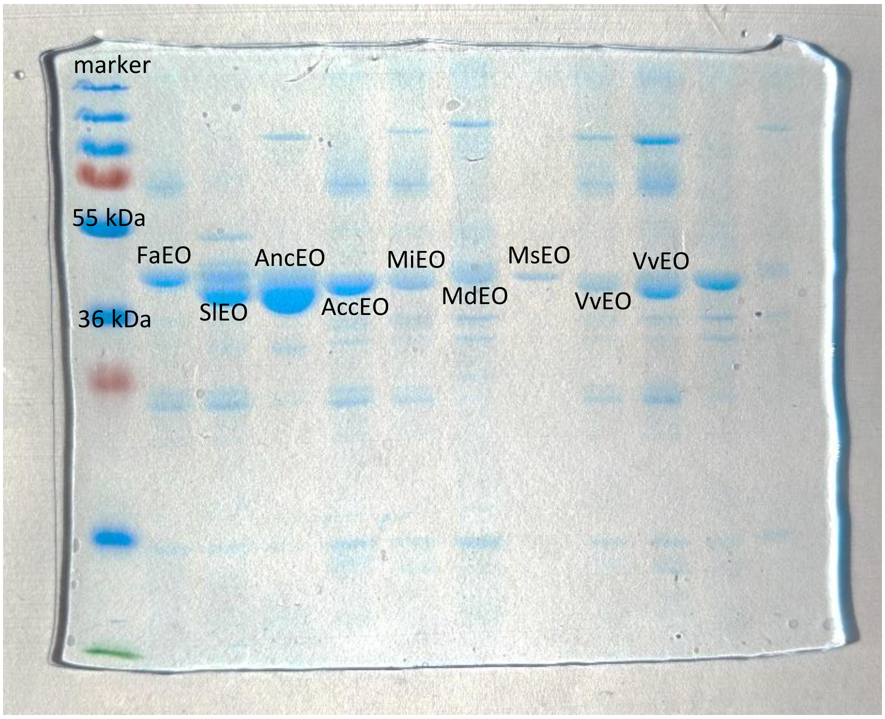

B

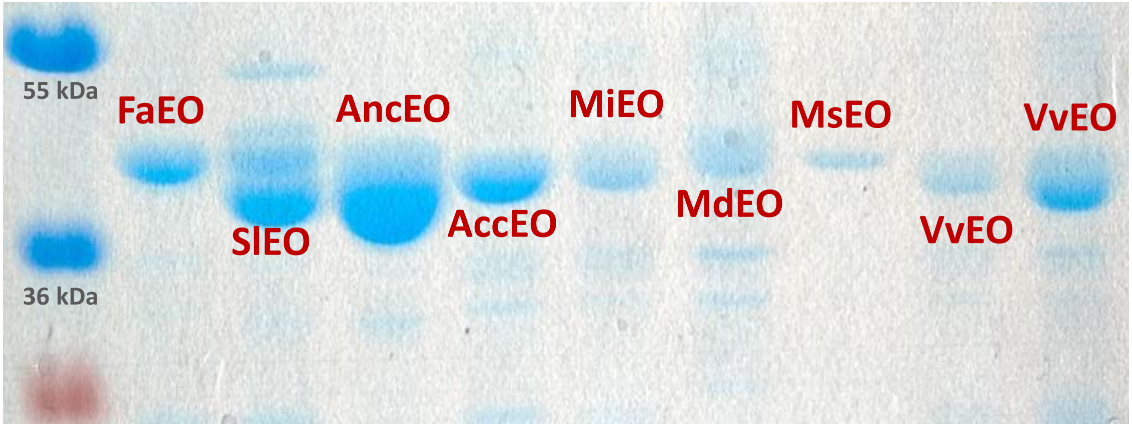

Figure S1. SDS-PAGE analysis of recombinant proteins. Recombinant enzymes analyzed include FaEO from *Fragaria x ananassa*, SlEO from *Solanum lycopersicon*, MiEO from *Mangifera indica*, VvEO from *Vitis vinifera*, AncEO from *Ananas comosus*, AccEO from *Actinidia chinensis*, MdEO from *Malus domestica*, and MsEO from *Malus sylvestris*. (**A**) Full SDS-PAGE gel. (**B**) Enlarged view of the relevant gel region

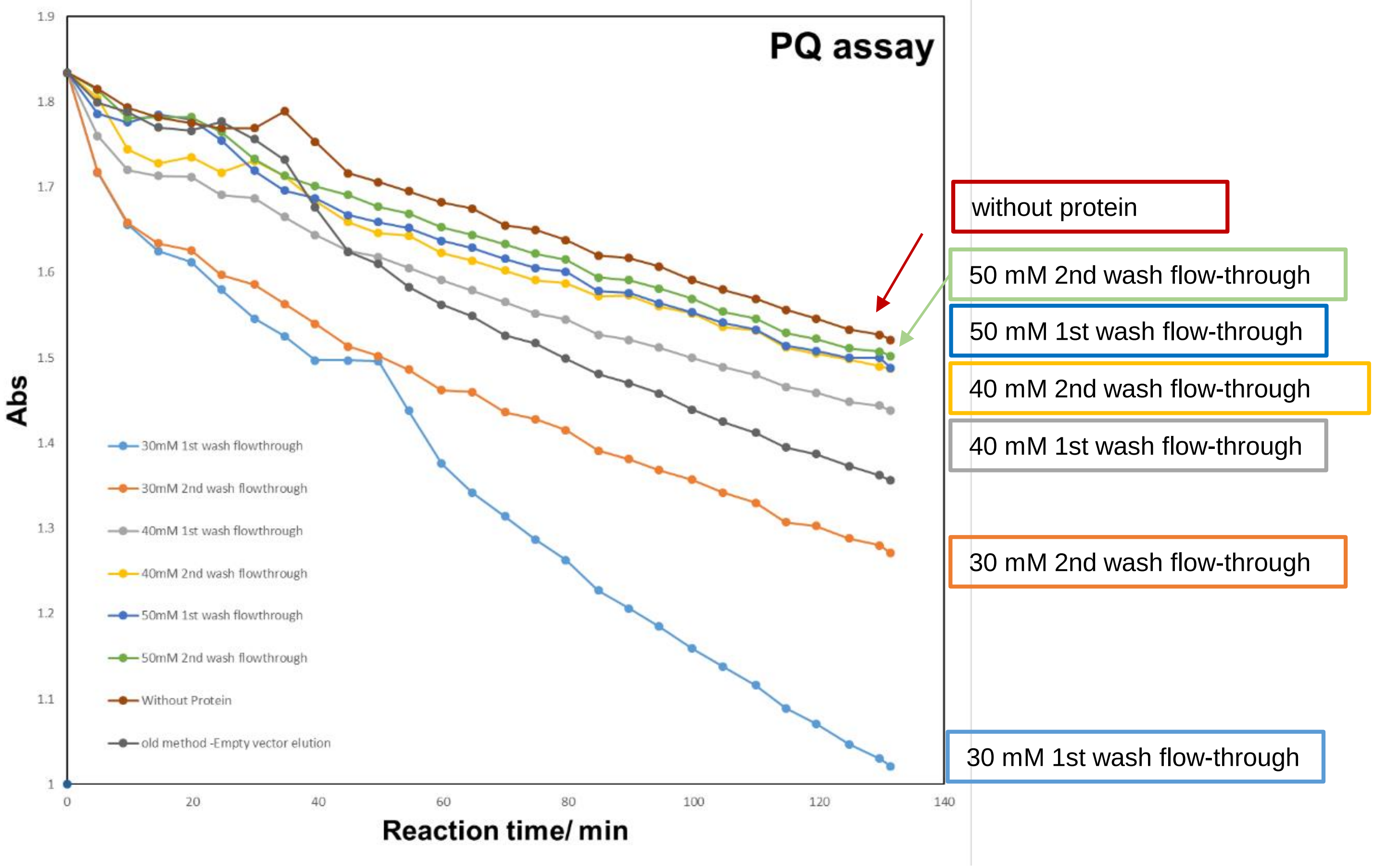

Figure S2. The enzyme activity from *E. coli* was effectively minimized. The use of wash buffers containing 30, 40, or 50 mM imidazole during affinity purification of *E. coli* containing empty vector were collected and ran PQ assay. The second 50 mM wash flow-through (green line) exhibited activity levels almost identical to the spontaneous chemical reaction (dark-red line), confirming that the washing procedure successfully eliminated endogenous PQ activity originating from *E. coli.*

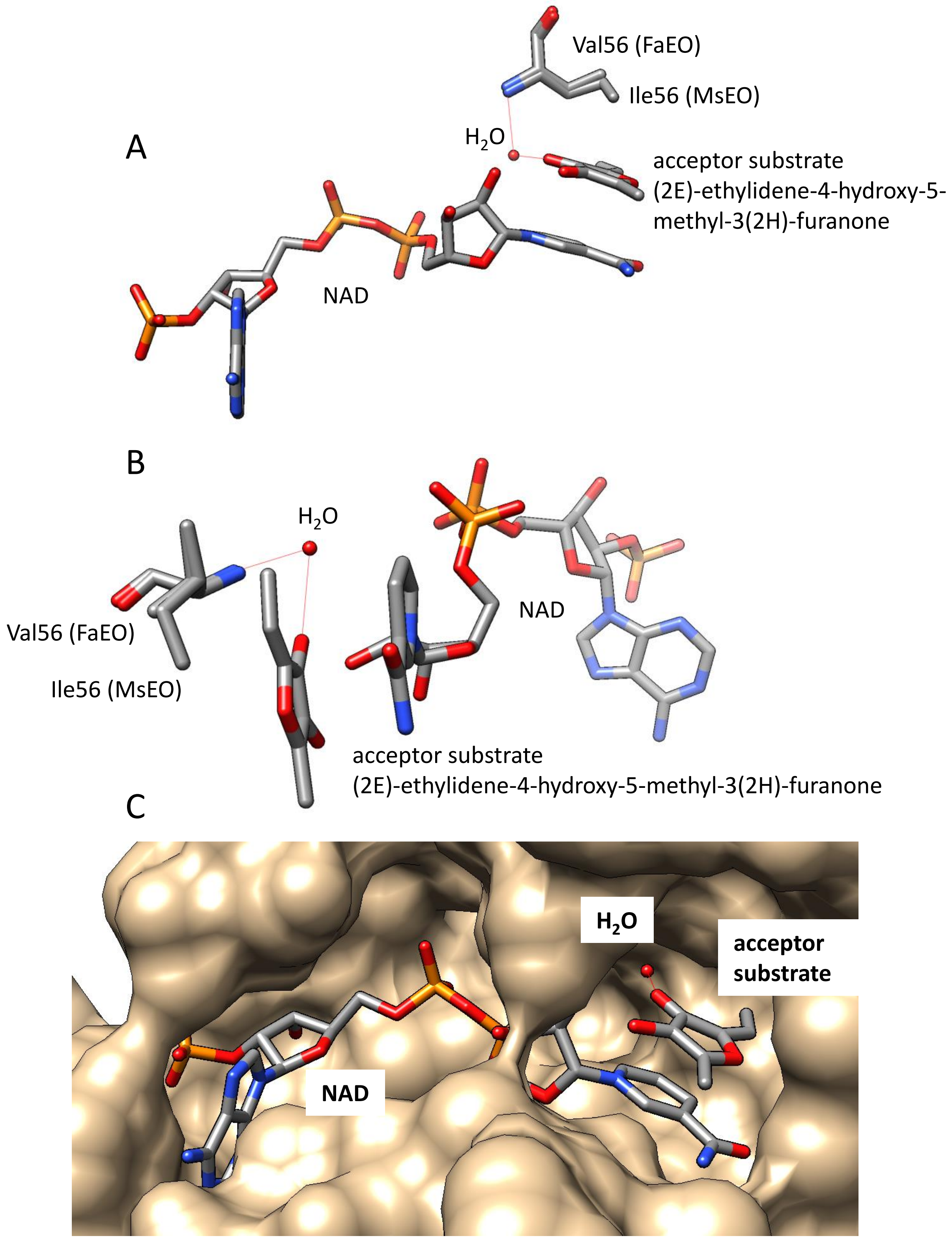

Figure S3. Substrate binding to FaEO. (**A**) Positioning of NAD and the surrogate acceptor substrate (2E)-ethylidene-4-hydroxy-5-methyl-3- (2H)-furanone in the 3D structure of FaEO (https://www.rcsb.org/; PDB: 4IDE). NAD and the acceptor substrate are shown as stick models; water molecules are depicted as red spheres. (**B**) Front view of the active-site region. (**C**) Close-up view of the substrates positioned within the active site.
